# Remarks on the Dysentery and Hepatitis of India

**Published:** 1847-01

**Authors:** 


### ART. IX.

*Remarks on the Dysentery and Hepatitis of India.* By E. A. PARKES, M.B., late Assistant Surgeon H. M. 84th Regiment.—*London,* 1846. 8vo, pp. 271.

OF the diseases incident to Europeans in tropical climates, there is perhaps none of more importance than dysentery, whether we consider the amount of mortality arising from it, or the permanently impaired health produced by alteration of structure in those who have laboured under it. In the army this is even more marked than in civil life, for the soldier, often necessarily exposed to the causes of disease, and frequently unwilling to submit to the necessary restraint when convalescent, suffers repeated relapses until the disease terminates either in death or in organic alterations of such a character as to render him permanently unfit for military service. Dysentery has consequently attracted much of the attention of the medical officers of the army, and many excellent works have been written upon it. There are still, however, many disputed points in regard to its pathology and treatment, especially in the frequent case of complication with other diseases; and much patient investigation and accurate observation will be requisite ere these can be settled on a satisfactory basis. The volume before us is a valuable contribution to this end, and is most creditable to the industry and talents of the author. It contains the result of his observations while serving in the 84th regiment in India, and professes less to be a systematic work on the diseases treated of, than an endeavour to elucidate various points connected with them about which differences of opinion prevail, and to draw attention to the composite nature of all chronic abdominal diseases. "Before long," the author remarks, "a different mode of describing the allied abdominal diseases will be necessitated by increasing knowledge. Then it will be found, that each disease, when fully formed, is but a developed and prominent part of a more general but partially latent affection. I am fully prepared to say, that a chronic affection of an abdominal organ never remains simple."

Dysentery. *Pathology.* Our author agrees with those who maintain the inflammatory nature of dysentery, but considers it to be peculiar in this respect "that ulceration of the large intestines occurs with great rapidity, and, except in one rare form, a case never presents true dysenteric symptoms without ulceration being present."—This does not arise from the intensity of the inflammation, for in slight cases where the patient has died suddenly from some other cause, "the proofs of inflammation, apart from ulceration, are often only just visible on post-mortem examination;" and in severe cases where extensive ulceration has been found, it frequently happens there has been during life very little constitutional disturbance. Dr. Parkes attributes the rapidity with which ulceration occurs, "to the glands of the mucous membrane being particularly implicated in the inflammatory action."

We shall endeavour to give, as concisely as possible, the statements advanced by him in support of his views, referring our readers to the work itself for the corroborative evidence contained in the post-mortem examinations. "1. There exists on the inner coat of the large intestines, a set of solitary glands peculiar to that particular mucous membrane." These are very different from the common mucous follicles, are hardly visible when the mucous membrane is healthy, but are enlarged and very evident at the commencement of dysentery. They have been noticed by various writers, but their relation to the dysenteric ulceration has not been pointed out, and they have been described as pustules.

"I have considered them not to be large mucous crypts for the following reasons. They present the appearance of round opaque bodies, without apparent orifice, imbedded in the mucous membrane, and even apparently attached to the submucous cellular tissue. In the early stage of dysentery, their contents are white, yellowish, and apparently thickened and starchy. They are sometimes streaked or striated on the surface, and bear on the summit, in some cases, a small black point, which looks like an orifice closed up. This is not, however, general or even common. Under the microscope, the mucous membrane around them presents the usual appearance of honeycomb cells. In a dysenteric case which has lasted two or three days, they are still more obvious. A minute vascular ring surrounds them, and they become prominent and a little hardened to the touch. In distribution, these glands appear equally numerous in the sigmoid flexure as in the cæcum; and on this account, I am disposed to regard them as perhaps the excreting organs of the colon." (p. 4.)

"2. Inflammation and ulceration of these glands constitute the earliest morbid change in tropical dysentery, and the process from the small ulcered gland to the irregular spreading ulcer, may be traced in every stage. The first alteration in the glands is an enlargement of them and a change in their contents. The contained substance becomes thicker, and now resembles flour and water in appearance and consistence. In all probability, this condition occurs every day, and giving rise to slight diarrhœa, relieves itself, and the glands return to their normal condition. . . . . . If, instead of thus relieving themselves by secretion, the glands continue enlarged for some time without being acutely inflamed, that appearance is presented which has been incorrectly compared by Ballingall and others to a variolous eruption. I have seen this several times, and the resemblance is about as great as might have been anticipated from the loose nature of the statement. . . If a greater degree of inflammation be present, the vessels around the gland become enlarged and conspicuous, and form a ring or halo spreading a short distance into the mucous membrane. This condition presents the earliest symptoms of dysentery, viz. slimy stools, increased in number without blood, causing perhaps slight griping and tenesmus when passed, and generally unattended by pain on pressure. Immediately after this, and in severe cases during the very first days, ulceration begins and is always denoted by slimy and gelatinous stools, streaked with blood, and attended by tormina, tenesmus, and pain on pressure, varying according to the *seat* of the disease and its *intensity*." (p. 6.)

Such are the views entertained by our author of the nature and seat of dysentery; and on these he founds his division of it, when uncomplicated, into four stages: 1. That of enlargement and commencing ulceration of solitary glands: this is the condition described in the preceding quotations. 2. Of complete and spreading ulceration. 3. Of cicatrization. 4. Of abortive cicatrization, commonly called chronic dysentery; a disease which is a resultant of continued subacute inflammation, and ulceration, combined with ineffectual efforts to produce the cicatrizing process.

"The second stage is characterized by the existence of ulcers, more or less numerous, of various shapes, sizes, and degrees of development, round, oblong, or irregular; if small and round, often with raised edges; if irregular, with flat and levelled edges. In the same case every form may be seen, from the commencing punctiform ulcer, to the complete large spreading ulcer with lymph on its surface in nodules or layers. This period is attended with various kinds of stools: first, these are slimy and gelatinous, becoming more and more bloody; then the stools become scanty, lymphy and shreddy, streaked with blood, or watery, muddy, and with sanious discharges. At a later period the stools become like the washings of meat, dark, and perhaps offensive. If the ulcers heal, the stools become generally, first, like lymph floating in an albuminous fluid; then yellow feculence, streaked with blood, is mixed with this, and then the stools recover gradually their healthy appearance." (p. 11.)

In describing the third stage, or that of cicatrization, Dr. Parkes combats the opinion, held by many writers, that ulceration exists only in the advanced stages of the disease, and refers to dissections of several cases, the subjects of which were cut off by coup de soleil at an early period of dysentery. The process of cicatrization of the ulcers is thus described by him:

"After a certain time, in dysentery, when the inflammation has diminished, lymph begins to be effused over the surface of the ulcer, and between the muscular fibres, if these form its floor. In an ulcer disposed to heal, the lymph is regularly diffused over the surface, forming a gelatinous-looking coating, which becomes gradually darker in colour, rises to a level with the edges of the ulcer and the surrounding membrane, and then slowly contracts, puckering to a greater or less extent the adjacent mucous membrane. After an uncertain length of time, varying from one to four months, the only marks by which it can be distinguished from normal mucous membrane are by its greater and darker vascularity, its greater smoothness and peculiar slightly glistening appearance, and by the slight contraction round it. In the majority of instances, however, the process is less regular than this; from some cause or other, greater quantities of lymph are deposited on some parts of the ulcer than on others, and hence results a granular or nodular appearance, which after a time disappears, and the false membrane becomes levelled and uniform. In some cases the lymph is deposited between the muscular fibres, apparently compressing these; the ulcer is then healed, that is to say, it will not spread, and no blood escapes from it. Afterwards on this compressed muscular floor lymph is slowly deposited." (p. 17.)

After recording a number of dissections (which appear to have been very carefully made) illustrative of the different stages of the disease, our author gives a table of the principal changes found in the other abdominal organs, in twenty-five cases which proved fatal in the garrison at Moulmein. That most frequently diseased was the liver, which in seven cases contained abscesses. These were found in the same number out of 39 cases treated by Dr. Innes, 84th regiment. Mr. Marshall states that they occurred in about the same proportion among the troops in Ceylon.

*Hepatic abscess.* Abscess of the liver has, by some writers, been considered to be always the primary affection, and the dysentery in these cases to be consecutive to, and arising from it. This, however, is held by some of the best authorities to be erroneous. Agreeing, in this respect, with Annesley, our author divides the cases of suppuration in the liver, into those in which the disease is primary, or antecedent to the dysentery, and those in which it is secondary, or consecutive to it; subdividing the latter into (*a*) declared, and (*b*) latent.

The connexion between hepatitis and dysentery, although generally recognized, has never been satisfactorily explained. In primary hepatitis it has been supposed by Annesley and others, that the dysentery is caused by the morbid state of the biliary secretion. Dr. Parkes dissents from this opinion, and hazards the conjecture that it is caused by the absence of secretion altogether. In support of this, he adduces the fact that in some cases "when hepatitis has terminated in partial suppuration, and bile is still secreted, although altered in appearance, there is no dysentery; whereas when, from extent or peculiar situation of abscess, no bile is secreted, dysentery appears to supervene."

Passing over the description of the symptoms of secondary hepatic abscess, with the illustrative cases and dissections, we come to the subject of diagnosis. This is universally admitted to be extremely difficult, from the obscurity of the symptoms, and their close resemblance to those of chronic dysentery, especially when that is complicated with functional disorder of the liver. We agree with our author that "it is only by a study of the phenomena, from day to day, that a correct diagnosis can be given;" but we do not think he has stated sufficiently clearly, or fully, the various points of difference which may assist in arriving at a correct conclusion. One point on which he differs from many writers deserves to be noticed:

"Cases in which pus has been absorbed and discharged with the urine have never been observed by me. I have seen thick, apparently purulent, deposits in the urine, and have heard them called 'decidedly purulent;' but these are mere collections of vesical mucus, of a particular kind; and exactly similar appearances are seen in pyelitis and catarrhal inflammation of the bladder, where there is no suspicion of pus being formed anywhere. These deposits are soluble with effervescence in acetic and nitric acids. No coagulation was ever observed from heat or nitric acid." (p. 98.)

This opinion corroborates that of Dr. Budd:

"It has been supposed by some medical men in India, that the pus in an abscess of the liver may be absorbed and eliminated *as pus* in the urine. But this notion is evidently erroneous. Pus-globules, from their large size, cannot directly enter the blood-vessels, or escape from them. The matter in the urine, supposed to be pus, was probably a deposit of phosphates. During the severe constitutional disorder that attends purulent phlebitis, there is often a sediment of this kind in the urine, having, to the naked eye, much the appearance of pus, but under the microscope, showing instead of pus-globules, beautiful phosphatic crystals." (Diseases of the Liver, p. 89.)

In endeavouring to account for the production of secondary hepatic abscess, our author disputes the opinion that it arises from the absorption of pus from the intestinal ulcers, or that it is a result of venous inflammation, or of an immense secretion of vitiated bile, and advances the hypothesis that it is caused by "a passage with the blood of those substances which, under ordinary circumstances, are excreted by the colon."

"When I examined," he remarks, "cases of consecutive hepatic abscess, I observed that the dysentery was general, though perhaps not very far advanced or very acute. The ulcers were sometimes small, and had healed early, but they were numerous and distributed universally over the mucous membrane of the large intestines; or if not everywhere ulcerated, all the glands were very large and hard to the touch. In other cases of dysentery, without hepatic abscess, the ulcers were perhaps very much larger, gangrenous, and altogether the colon may have appeared more diseased, but still there were clear spaces of undiseased mucous membrane. I therefore at length came to the conclusion, that the type of dysentery generally associated with the consecutive abscess is one in which there is universality of affection, with or without a high degree of intensity of inflammation. . . . . . . . . Is it not an allowable hypothesis that the normal action of part of the mucous membrane will prevent abscess, by excreting some undetermined ingredient, which in the other case (where the affection of the membrane is universal) must be circulated with the blood, and then by its effect on the liver produces suppuration in that allied organ? I state this as an hypothesis, that is, as an imaginary arrangement of facts which is to be tested by experience. The facts are, the intimate connexion of dysentery and abscess, which is undoubted, and the universality of affection of the colonic solitary glands, in secondary hepatic abscess—a fact which requires further observation to confirm it." (p. 117.)

Adverting to the great rarity of hepatic abscess among the natives of India, which our author considers to afford very strong évidence against the existing theories, he observes:

"According to the view I have taken, the difference between Asiatics and Europeans may be attributed to the difference in food, and consequent difference in the composition of blood and excretions, and to the difference of the skin, which in the former nations excretes more oily and carbonaceous perspiration." (p. 119.)

*Complications.* Dysentery is occasionally complicated with scurvy or purpura, and proves a most formidable disease. The most important differences between this and the simple form are, that the "ileum participates in the disease, and is sometimes more affected than the large intestine; the cæcal and colonic solitary glands ulcerate in the usual way, but the lymph thrown out does not circumscribe the ulcers; perforations are common; the intermediate mucous membrane is darkly vascular, and often softened, and appears to effuse blood even when unulcerated." This form of the disease will not bear depletion or active purging, and a few grains of calomel will often produce severe ptyalism. Dr. Parkes has found creosote, in combination with opium, very useful in such cases, after the acute stage has passed.

*Causes.* The exciting causes of dysentery are divided by our author into four classes: 1. All acrid agents, whether produced by irritating ingesta or secretions. 2. Suppression of secretions rapidly accomplished. 3. Epidemic states of the atmosphere. 4. Alterations in the blood, effected by some peculiar and at present unknown changes in the process of assimilation (scurvy, purpura, &c.)

*Treatment.* The indications of treatment are: 1. To subdue the inflammation of the solitary glands, and of the ulcers, when these are formed, as is always the case after the first few days. This comprises: *a*, the removal of the causes; *b*, the removal of morbid secretions; *c*, the restoration of the functions of the, liver, skin, and kidneys. 2. To assist the healing of the ulcers, when the ulceration has been arrested. The measures recommended to fulfil the first indication are briefly summed up as follows:

"1. Very free depletion, to arrest the progress of an acute inflammation. 2. Oleaginous purgatives, with opium, to remove secretions. 3. Opium, to allay tormina, and diminish the excess of nervous sensibility, which is one link in the inflammatory process; the combination of blue pill and ipecacuan with the opium seems to increase its powers. 4. Occasional production of salivation." (p. 146.)

With regard to the treatment of dysentery by salivation, formerly the almost universal practice, Dr. Parkes, after stating his objections in full, thus sums up with, we think, a less forcible protest against the meddlesome mischief than he was entitled to make:

"Considering that recovery is certainly slower with this treatment than with the depletion and alterative plan, that, in India, a scorbutic taint or an adynamic habit of body often accompanies dysentery in European soldiers, and remembering the great difficulty of limiting the effects of mercury, when rapidly administered, to a moderate action, it must be confessed that the utility in dysentery of this very powerful remedy has been rated too highly by some of its supporters." (p. 144.)

In discussing the means to be employed "to assist the healing of the ulcers" in chronic dysentery, Dr. Parkes divides the cases into four classes:

"1. Immediately on ulceration being partly checked, the reparative process begins, and vast quantities of lymph are thrown out upon the ulcers and between the intestinal tunics. If to this we have superadded, from time to time, attempts at fresh ulceration, followed as before by effusion of lymph, we get a bad form of chronic dysentery, in which the intestine becomes immensely thickened, and, as a consequence, partially lessened in caliber in different parts of its course, and enormous masses of nodular or granular lymph are effused on the mucous membrane, and the ulcers, wholly or partially concealing these parts. 2. Or, subacute dysentery being unchecked and becoming chronic, we get a state of parts in which all the ulcers may be healed, and no fresh ulceration going on, but the coats being densely thickened, and the functions of the mucous membrane completely interrupted, we have a long continued and exceedingly intractable form of lienteric dysentery; in the latter stage of which, if the thickening be universal, hepatic abscess may probably supervene." (p. 149.)

The treatment in these two forms should consist in local depletion, with a strict farinaceous diet, followed by the cautious administration of mercury till a slight action on the gums is produced; counter-irritation; and afterwards, if necessary, the nitrate of silver, or the nitro-muriatic acid.

"3. Another form of chronic dysentery, and a very common one, is that in which the original attack has been almost chronic from the first, or at any rate not severe, and in which the glands get hypertrophied and slightly ulcerated, and a small quantity of lymph thickens at intervals the coats of the large intestine."

In this form the stools, instead of being feculent, loose, lienteric, or occasionally serous, are lymphy, fatty, dark, viscid, or variegated; or these varieties alternate with the former.

"4. Another form of chronic dysentery is more passive, following colonitis or erythematous dysentery, and consisting of pale ulcers, with the muscular fibres for their floors, prevented from spreading by effusion of lymph, which is yet not effused in sufficient quantity to heal them." (p. 150.)

In these cases metallic astringents are to be given, as the sulphates of copper, zinc, and iron, followed up by tonics combined with alteratives. In all cases the diet should be carefully attended to, being as much as possible restricted to farinaceous food, while beer, wine, and other stimulants should be interdicted.

Hepatitis. Having discussed the subject of dysentery and secondary hepatic abscess, Dr. Parkes proceeds to the consideration of hepatitis. This disease is one of great importance to the military surgeon in India, as giving rise to a large amount of ultimate inefficiency. Of 41* cases of true hepatitis, which our author has tabulated, 6 died, 15 were invalided, 3 relieved, but not cured, 12 cured, and 5 remained under treatment when the table was drawn up. Thus it appears that upwards of one half of those attacked were ultimately lost to the service by death or invaliding.

*Causes.* Passing over the symptoms of hepatitis, and the various forms it assumes, we give a portion from our author's most important observations on its causes. We feel well satisfied of their justice, and are convinced that a little attention by the authorities to the improvement of the diet of the soldier would, especially in tropical climates, be attended with most beneficial results. Referring to the most common form of liver disease in India, "gastro-duodenal hepatitis," Dr. Parkes remarks:

"The diet of European soldiers in India, varying necessarily at different places, is, as a general rule, far too rich and stimulating. Hot curries, carelessly made by native cooks, are used several times every week for dinner; and vegetables are in many places scarce or of indifferent quality. Soldiers often refer the origin of their complaint at once to their diet, and, to my own knowledge, many men have supplied the place of the curries by rations purchased out of their own scanty funds. It often happens that an European regiment quartered with one or two companies of English artillery will show a much greater per centage of sickness: the habits of both corps are the same with one exception; artillerymen, living in small bodies, are easily looked after by their officers, and they are generally more careful about their diet. Again, married men who are not in a mess are always more exempt from both dysentery and hepatitis than single. If this is not attributable to their food being better cooked, the circumstance is inexplicable. It is an extraordinary thing that out of 150 married men in the 84th regiment, only two died during a tropical service of 30 months, while in the same period, the mortality among the single men was above nine per cent. The two deaths referred to were from phthisis and from delirium tremens. Some influence may be given to the habits of married men being more regular than those of single men, but in a small station where little debauchery goes on, the influence cannot be great.

* The number of cases is stated to be 42, but there are only 41 given in the table.

"A supervision of the whole system of diet among European troops—not as regards commissariat supplies, which are generally excellent, but as respects the cooking of these, and the time of meals, the encouragement of teetotal societies by every allowable means, and the formation of day and night guards, differently clothed to prevent the effects of the great daily thermometrical range of some Indian stations—are measures which I am convinced would at once reduce the list of duodenal hepatitis, and would probably even diminish the number of cases of dysenteric, febrile, and primary hepatitis." (p. 227.)

After discussing at some length the various exciting causes which have been assigned by different writers on this subject, our author thus briefly sums them up:

"1. Hepatitis, in many cases, is clearly caused by dysentery and remittent fever. 2. The influence of bad or improper food, spirituous liquors, &c., in causing hepatitis is probably to be explained by their effect on the gastro-duodenal mucous membrane. 3. Heat alone has not been proved to be a cause of primary hepatitis. The hot stations in the Madras Presidency are the healthiest; for instance, Bellary and Trichinopoly. 4. But as a collateral agent, heat has a great effect; it increases secretions, as of the skin, or alters them, as of the kidneys. To these increases and alterations by themselves the system seems to accommodate itself, but not to rapid transitions in them. There appears ground for believing that secretions increased by great temperature, and then suddenly suppressed or lessened by the abrupt supervention of another atmospheric condition, really have an influence, unexplained but decided, upon the liver in particular......5. The doctrine of pulmonary and hepatic antagonism by which the liver is supposed to be called upon, in hot climates, to excrete carbon, which in cold countries the lungs give off, has not been proved, as far as my knowledge goes, and at present is merely an ingenious conjecture." (p. 234.)

We do not intend to follow our author in his remarks on the pathology and treatment of hepatitis, agreeing with him that the former is very imperfectly known, "and that many of the most important means of studying it are at present little understood; when chemical organic analysis can be more easily performed, several points which can now only be guessed at will be cleared up. Till this be the case, the treatment will remain, as it is now, vague and empirical, and the direct opposite of the certain and decisive method of treating acute dysentery, to which a correct knowledge of the morbid anatomy of this disease has led us." (p. 248.) We shall, however, submit our author's conclusions as to the existence of urea in the urine of patients labouring under hepatitis, a question which is still disputed:

"In diseases in which the secretion of bile is stopped, as in great primary or consecutive abscesses, where there is no jaundice, no bile in the stools, and none in the gall-bladder after death, we find the secretion of urea to be also arrested or nearly so. I draw this conclusion—first, from the lowness of the specific gravity; secondly, from the pale colour; and, thirdly, from the impossibility of getting nitrate of urea in the usual way.

"In that universal chronic dysentery where there are yellow liquid stools without bile, and in which there is no jaundice, the urea is also diminished, as in the former case. The liver seems not to secrete bile sometimes for days together, or even weeks. Abscess often supervenes. In duodenal hepatitis, without jaundice, and where bile is secreted, though perhaps in less quantity than natural, the urine is high coloured, and of usual or high specific gravity, and the urea is apparently in normal proportion, or, at any rate, not lessened. In acute gastro-duodenal dyspepsia, with the liver acting more than usual, judging from the copious and dark or orange-coloured stools, and, judging from percussion, also enlarged, probably from biliary as well as venous congestion, the urine is of great specific gravity, so that the urea is probably secreted in undue proportion. In duodenitis with jaundice, where abundance of bile is secreted, the urine is loaded with urates, and probably with urea, as well as with the colouring matter of the bile. So that there really does seem to be a connexion between the secretion of the kidneys and liver, by means of which we may hope in time to acquire some knowledge of the condition of the latter organ. Whenever the secretion of bile is stopped there appears to be a diminution in the quantity of urea separated by the kidneys." (p. 252.)

This is a subject well deserving of further investigation. Twenty-five years have elapsed since the non-existence of urea in hepatitis was called in question by Dr. Prout and Dr. Davy, and yet nothing has been done to settle the point!

In concluding our remarks on the volume before us, we may repeat that we consider it very creditable to the author's talents and industry. It is a work adapted for the practitioner rather than the student, and should be suggestive to him of many useful reflections. We trust those who have the opportunity will test the facts adduced by Dr. Parkes in support of his hypotheses, and will follow up his observations to verify or overthrow them. Much also remains to be done by the aid of microscopic investigation, and still more of chemistry. To these we would specially recommend increased attention, and at the same time caution our readers not to neglect the means of investigation at present employed.

"I am not of the opinion of those," our author judiciously observes, "who believe that the old paths of investigation—the study of symptoms and of post-mortem appearances—are now exhausted. If this be the case, how is it that the morbid anatomy of dysentery has not, to my knowledge, hitherto been fully described? How is it that in cholera, in spite of ample opportunities, every new writer discovers something that his predecessors have overlooked? How is it that the most important renal diseases have only been described within the last few years? Because new modes of investigation have been opened to us, why should we give up the old ones?"

We take our leave of this volume with sentiments of great respect for its author. We feel assured that we shall again meet him in the field of medical literature, which the present work proves him so well qualified to cultivate and enrich. We see here combined quickness of observation, clearness of conception, ingenuity of speculation, and a calmness and soundness of judgment, unusual in a young writer, and not too common in those of maturer years. The medical service of the army has cause to regret the early departure from its ranks of an officer so well qualified to add to its already high reputation; but we hope the new sphere of duties on which Dr. Parkes has entered, may afford to himself and to the profession sufficient compensation for the loss.

